# Neighborhood Disadvantage and Cardiovascular Mortality Among Colorectal Cancer Survivors

**DOI:** 10.3390/cancers17233782

**Published:** 2025-11-26

**Authors:** Nimish Valvi, Matthew Groenewold, Krista Terracina, Himanshi Verma, Pratibha Shrestha, Kathryn E. Hitchcock, Dejana Braithwaite, Shama D. Karanth

**Affiliations:** 1 Department of Nutrition and Health Science, Ball State University, Muncie, IN 47306, USA; nimish.valvi@bsu.edu (N.V.); matthew.groenewold@bsu.edu (M.G.); 2 Department of Surgery, University of Florida College of Medicine, Gainesville, FL 32610, USA; krista.terracina@surgery.ufl.edu (K.T.); hverma@miami.edu (H.V.); pratibha.shrestha@surgery.ufl.edu (P.S.); dbraithwaite@surgery.ufl.edu (D.B.); 3 Department of Radiation Oncology, University of Florida, Gainesville, FL 32610, USA; 4 University of Florida Health Cancer Center, Gainesville, FL 32608, USA

**Keywords:** socioeconomic status, geographic health disparities, comorbidity

## Abstract

**Background:** Cardiovascular disease (CVD) is a common cause of death among colorectal cancer (CRC) patients. We examined whether neighborhood disadvantage is associated with CVD mortality in CRC patients. **Methods:** Data were obtained from the Surveillance, Epidemiology, and End Results (SEER) program for primary CRC patients diagnosed between 2006 and 2017. Neighborhood disadvantage was measured using the quintiles of the Yost Index, a socioeconomic composite measure. Cause-specific mortality (CVD-specific and CRC-specific mortality) was evaluated using a competing risk cause-specific hazard model, controlling for demographic and clinical covariates. Cumulative incidence function (CIF) and restricted mean survival time (RMST) analyses were performed to provide complementary estimates of absolute risk and survival differences. **Results:** The study included 316,549 patients with CRC. Cancer-specific mortality was the leading cause of death (62.1%), while CVD accounted for 9.6% of deaths. Multivariable competing risk Cox regression showed that the lowest-SES neighborhoods (Group 1) had a higher CVD-specific mortality (HR, 1.39; 95% CI, 1.30–1.48; *p* < 0.001) compared to the highest-SES neighborhoods (Group 5). RMST and CIF analyses revealed a similar dose–response pattern, with progressively higher CVD mortality associated with increasing levels of neighborhood disadvantage. Effect modification analyses indicated stronger associations in older patients and men, but no modifications by race. **Conclusions:** Among CRC patients, residing in disadvantaged neighborhoods was independently associated with higher CVD mortality, suggesting the importance of addressing cardiovascular risk in disadvantaged populations.

## Simple Summary

This study examined whether where a person lives influences their risk of dying from heart disease (CVD) when they also have colon or rectal cancer (CRC). Using data from 316,549 CRC patients, we found that over a median follow-up of 39 months, 146,206 patients died. Although cancer was the leading cause of death (62.1%), heart disease accounted for a substantial portion (9.6%) of deaths. Patients living in the poorest neighborhoods had a 39% higher risk of dying specifically from heart disease compared to those in the affluent neighborhoods. This increased risk was evident even after accounting for the patient’s age, tumor stage, and other individual health factors. The link between neighborhood poverty and heart disease death was stronger for older patients and men. This suggests that factors related to living environment, such as access to quality healthcare, comorbidities, and lifestyle habits, may play a role in contributing to differences in survival outcomes.

## 1. Introduction

Colorectal cancer (CRC) is the fourth most common cancer and the second leading cause of cancer-related death in the United States, with an estimated 154,270 new cases and 52,900 deaths projected in 2025 [1,2,3]. Advances in cancer treatment have markedly improved cancer-specific survival, but cardiovascular disease (CVD) has become a leading cause of non-cancer mortality among CRC survivors [4,5,6,7,8]. This heightened risk is due in part to the cardiotoxic effects of certain cancer therapies and shared risk factors such as obesity, hypertension, diabetes, smoking, diet, physical activity, and social determinants of health [9,10,11,12]. CRC survivors are two to four times more likely to develop CVD compared to the general population [7]. Population-based studies have consistently shown that CVD is the leading cause of non-cancer death in CRC survivors [7,9]. In a multi-registry study, 42% of non-cancer deaths among CRC survivors were attributable to CVD [9]. Another study reported that 13.7% of CRC survivors died from CVD, compared with an average of 11.3% across all cancer types [13]. This places CRC among the top five cancer types with the highest proportion of CVD-related deaths [13]. Findings from these studies underscore the significance of survivorship research and subsequent interventions aimed at reducing CVD risk in CRC patients.

Neighborhood disadvantage, characterized by higher poverty, unemployment, and lower educational attainment [14], has been linked to a greater incidence of CVD in the general population [15,16,17]. Adverse neighborhood environments may lead to chronic exposure to stressors such as housing instability, limited access to health care resources, and social disorder [16]. Among CRC survivors, these stressors may compound with treatment-related cardiovascular toxicities and a high chronic disease burden, but their influence on cardiovascular outcomes in this population is not well understood [18,19].

This represents a critical gap in CRC survivorship research, as failure to consider neighborhood disadvantage may exacerbate existing disparities and limit the effectiveness of proposed interventions. CRC survivors may be particularly vulnerable due to cumulative treatment exposures and pre-existing comorbidities [10]. Understanding the relationship between neighborhood context and CVD mortality is essential for reducing disparities and optimizing survivorship care [20]. This study addresses this gap by examining the associations of neighborhood disadvantage, as measured by the Yost Index [21,22,23], with CVD-specific mortality after adjustment for factors historically associated with worse outcomes (e.g., Black race, later stage at diagnosis, and lack of treatment) [24,25,26]. Our objective was to determine whether increasing levels of neighborhood disadvantage are independently associated with higher CVD-specific mortality among CRC survivors. We hypothesized that CRC survivors residing in disadvantaged areas would be independently associated with higher CVD-specific mortality, even after accounting for these factors. We also explored whether this association varied by age, sex, and race.

## 2. Materials and Methods

### 2.1. Population and Data

Data for this study was drawn from the publicly available Surveillance, Epidemiology, and End Results (SEER) database of the National Cancer Institute [27]. SEER*Stat software version 8.4.5 (https://seer.cancer.gov/seerstat/, accessed on 10 February 2024) was used to access the SEER Research Plus Data (Specialized with Census Tract SES/Rurality), which encompasses 18 cancer registries (excluding Alaska) from the November 2020 submission, covering the years 2006–2018 [27]. This dataset includes approximately 28% of the U.S. population aged 20 years or older who were diagnosed with a primary colorectal malignancy (ICD codes C18–C20) between 2006 and 2017. Cases diagnosed in 2018 were excluded to ensure at least one year of follow-up for survival analyses. This study adhered to the Strengthening the Reporting of Observational Studies in Epidemiology (STROBE) reporting guidelines [28]. This study was exempt from review by the Institutional Review Board of the University of Florida because the analysis was based on publicly available data.

### 2.2. Primary Exposure

Neighborhood disadvantage was assessed using the census tract–level Yost Index, as defined by Yost et al. [21]. Prior studies have demonstrated the validity of the Yost Index in cancer outcomes [23,29,30]. Higher scores correspond to higher socioeconomic status (SES), with lower scores indicating greater neighborhood disadvantage [21]. The Yost Index is based on geocoded patient-level, location-based, and census-tract level data that incorporates seven measures: education index, median household income, percent unemployed, percent below 150% of the poverty line, percent working class, median house value, and median rent [21]. Census tract-level data is preferable to county-level SES measurements because the smaller tracts offer greater resolution and specificity in characterizing socioeconomic status [31]. Index scores were categorized into quintiles, with group 1 representing the most disadvantaged neighborhoods (lowest SES) and group 5 representing the least disadvantaged (highest SES), each containing approximately equal proportions of the population [21]. Neighborhood disadvantage classification was assigned to all cancer cases based on the census tract of residence at the time of diagnosis.

### 2.3. Outcome

SEER determines mortality through state and national vital records, with causes of death assigned using death certificates and ICD codes [27]. The primary outcome of this study was CVD-specific mortality, defined as the time from the date of CRC diagnosis to death from CVD. The CVD-specific deaths as recorded in the SEER database included deaths due to six specific causes: diseases of the heart, hypertension without heart disease, cerebrovascular disease, atherosclerosis, aortic aneurysm and dissection, and other diseases of the arteries, arterioles, and capillaries. The patients with the following CVD mortality were identified using ICD-10 codes I00-99. Secondary outcomes included all-cause mortality and CRC–specific mortality. Patients who were alive at the end of the study or lost to follow-up were considered censored.

### 2.4. Covariates

Covariates were selected based on existing literature and subject-matter expertise to assess the association between neighborhood disadvantage and survival [7,9,24]. Included covariates were: age at diagnosis (categorized as 20–49, 50–64, 65–74, and 75+ years); race and ethnicity (non-Hispanic [NH] White, NH-Black, NH-Asian/Pacific Islander [Asian/PI], and Hispanic [all races]); marital status (married/partnered vs. never married/divorced/separated/widowed/unknown); and year of diagnosis (2006–2008, 2009–2011, 2012–2014, 2015–2017). SEER summary stage was classified as localized, regional (including regional by direct extension, regional lymph nodes, both direct extension and lymph node involvement, or not otherwise specified), distant (distant site[s]/node[s] involved), and unstaged/unknown. Tumor location was defined as colon (right, left), rectum, or unspecified. Tumor grade was categorized as well-differentiated, moderately differentiated, poorly differentiated, undifferentiated, anaplastic, or unknown. Treatment receipt was defined as surgery (yes/no), radiation (yes/no), and chemotherapy (yes/no).

### 2.5. Statistical Analysis

Frequencies were calculated for categorical variables, and the median with interquartile range (IQR) was reported for continuous variables. Missing data for categorical variables were replaced with the level of “unknown,” whereas no missing data were observed for continuous variables. Overall survival was analyzed using Kaplan–Meier curves and the log-rank test. Cumulative incidence function (CIF) curves [32] using the Fine-Gray subdistribution hazard model, were generated to describe the incidence of CVD-specific mortality, accounting for competing risks from CRC-specific and other causes of death. The analysis was stratified by Yost Index groups, allowing for visual comparison of the cumulative incidence of CVD mortality across the socioeconomic gradient throughout the follow-up period. Adjusted hazard ratios (aHRs) and corresponding 95% confidence intervals (CIs) were estimated using Cox proportional hazards regression to assess overall mortality. Proportional hazards assumptions were formally evaluated for all covariates using cumulative martingale residuals and the supremum test (ASSESS statement, SAS), and no violations were detected [33,34]. To account for competing risks, we used the cause-specific Cox proportional hazards model [33] to estimate aHRs (95% CIs) for CVD-specific and CRC-specific mortality [9]. This approach allows deaths from other causes to be treated as censored at their time of occurrence, enabling us to assess the association between covariates and the instantaneous risk of each cause-specific death. In the cause-specific models, deaths due to other causes were censored at their time of occurrence. For the CVD-specific model, cancer-specific deaths were considered a competing event [33]. Covariates included in the model were age, sex, race/ethnicity, marital status, year of diagnosis, stage, grade, tumor location, surgery, chemotherapy, and radiotherapy. Effect modification was assessed using the likelihood ratio test; the interaction between effect modification and age, sex, and race/ethnicity was explored with the Yost Index groups. An interaction was considered significant if the *p*-value for the interaction term was <0.05, and assessment for final inclusion in the model was based on between-model comparisons using the likelihood ratio test: only models that offer significantly better goodness of fit than the original model were selected. To complement hazard ratio estimates, we report the differences in the restricted mean survival time (RMST) between Yost Index quintiles at 60 months, providing an interpretable measure of average survival time across socioeconomic strata [35]. Statistical significance was determined using two-sided tests with *p* < 0.05. All analyses were performed using SAS version 9.4 (Cary, NC, USA).

## 3. Results

### 3.1. Population Characteristics

Overall, 316,549 patients with colorectal cancer met the study criteria (Figure S1). Patients were classified according to Yost index quintiles: 25.0% (n = 79,082) resided in the most advantaged neighborhoods (group 5, highest SES), while 17.2% (n = 54,593) lived in the most disadvantaged neighborhoods (group 1, lowest SES). In the most advantaged neighborhood, the highest proportion of non-Hispanic (NH) White patients was observed (58,999 patients [74.6%]), whereas the most disadvantaged neighborhoods had the highest proportion of NH-Black individuals (16,935 patients [31.0%]). Individuals living in the most disadvantaged neighborhoods (group 1) had the highest proportion of younger patients (<65 years), divorced, separated, or single status, and a lower median survival time of 36 months, with an IQR of 13–74 months (Table 1).

### 3.2. Survival Analysis

**Figure 1:**
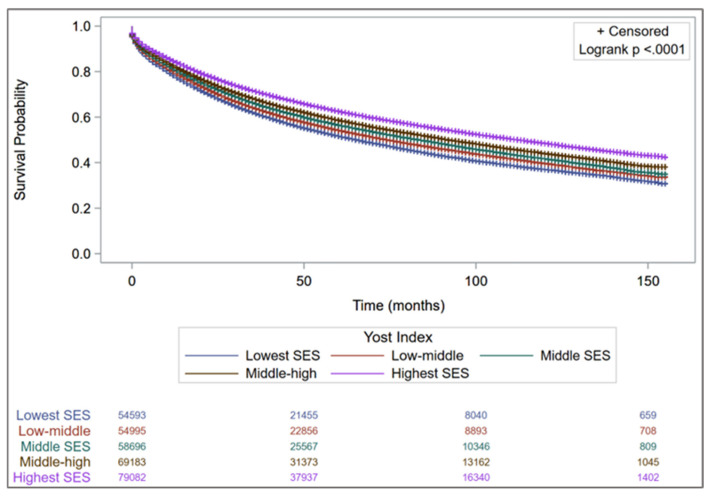
Kaplan–Meier Curves for Overall Survival by Yost Index Quintiles.

There were 146,206 deaths during the study period (2006–2017), including 90,801 (62.1%) CRC-specific deaths, 13,983 (9.6%) CVD-specific deaths, and 41,422 (28.3%) from other causes (Supplementary Figure S1). On Kaplan–Meier analysis, overall survival differed significantly across Yost Index groups (*p* < 0.0001; Figure 1).

**Figure 2:**
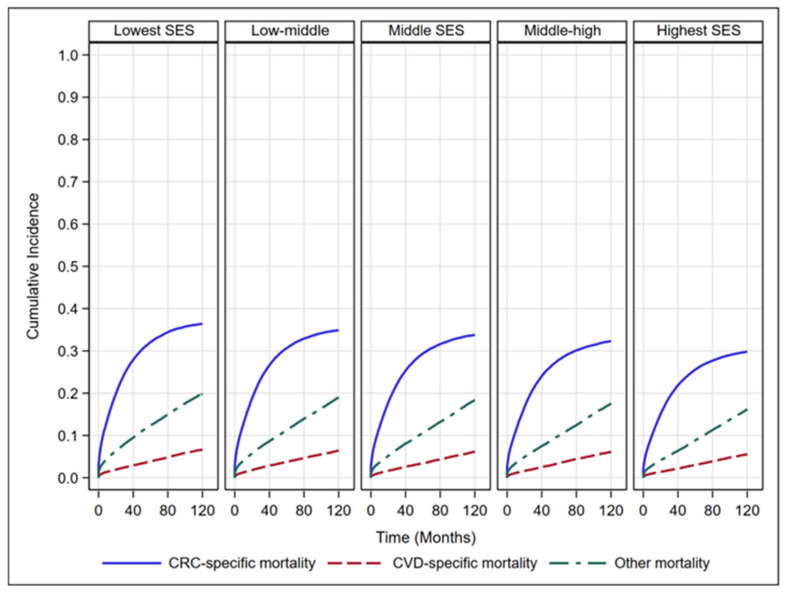
Cumulative Incidence of Mortality by Yost Index Quintiles.

The 10-year cumulative incidence of CVD-specific mortality across all Yost Index groups (Gray’s test, *p* < 0.001), accounting for competing risks from CRC-specific and other causes of death (Figure 2). The unadjusted CIFs revealed a consistent SES gradient for CVD-specific, CRC-specific mortality, and death due to other causes. Individuals in the lowest SES group experienced the highest cumulative incidence of all three outcomes. In contrast, the highest SES group exhibited the lowest incidence of all three outcomes. The curves demonstrated a clear “dose–response” relationship, with cumulative incidence decreasing progressively from the lowest to the highest SES category over the follow-up period.

Note: Each panel represents a distinct cause of death: cardiovascular disease (CVD), colorectal cancer (CRC), or other causes by Yost Index quintiles. Curves depict the probability of death from the specified cause over time, accounting for competing risks. Data is drawn from SEER for the years 2006–2018. Higher Yost index quintiles indicate greater neighborhood advantage.

Compared with the highest SES quintile group, lower SES quintile groups were associated with progressively higher mortality risks after adjustment for potential confounders (Table 2). For overall mortality, adjusted hazard ratios increased across SES quintile groups: 1.10 (95% CI, 1.06–1.14) in Group 4, 1.18 (95% CI, 1.14–1.21) in Group 3, 1.23 (95% CI, 1.20–1.26) in Group 2, and 1.31 (95% CI, 1.28–1.33) in Group 1 (lowest SES) (*p*-trend < 0.001). In the cause-specific analysis for CVD mortality, the aHRs also increased with lower SES groups, ranging from 1.07 (95% CI, 0.94–1.22) in Group 4 to 1.39 (95% CI, 1.30–1.48) in Group 1 (*p*-trend < 0.001), indicating the strongest relative impact of neighborhood disadvantage on CVD outcomes. For cancer-specific mortality, a clear gradient was also observed, with aHRs ranging from 1.09 (95% CI, 1.06–1.11) in Group 4 to 1.24 (95% CI, 1.18–1.30) in Group 1 (*p*-trend < 0.001). These results demonstrate a graded inverse association between neighborhood SES and mortality, with the most disadvantaged neighborhoods exhibiting the highest risk across all outcomes, particularly for all-cause and CVD mortality.

The RMSTs were calculated over a 60-month horizon and demonstrated progressively shorter survival with increasing neighborhood disadvantage across SES quintiles (Table 3). Group 5 (highest SES) had the longest RMST of 45.5 months (95% CI, 45.3–45.6). Compared to this reference, RMST decreased incrementally in more disadvantaged neighborhoods, with Group 1 showing the most significant reduction of 5.0 months (95% CI, −5.2–4.7; *p* < 0.0001). All pairwise differences between groups were statistically significant (*p* < 0.0001), indicating a clear socioeconomic gradient in survival.

### 3.3. Effect Modification

Significant effect modification by neighborhood SES was observed across demographic subgroups (Supplementary Tables S1–S3). CVD mortality increased sharply with age and was consistently higher among men, with the steepest gradients in the most disadvantaged neighborhoods (all *p* for interaction < 0.0001). In contrast, racial and ethnic differences in CVD mortality were less pronounced (*p* interaction = 0.62); however, Asian/Pacific Islander and Hispanic individuals had a lower risk of CVD mortality than White individuals across all SES quintiles. Males consistently experienced higher CVD-specific mortality than females across all SES quintiles, indicating sex-based differences in the relationship between neighborhood disadvantage and cardiovascular risk. For all-cause and CRC mortality, SES-related differences in mortality were also evident, with stronger gradients among older adults and men.

## 4. Discussion

In this large, population-based study, we found that greater neighborhood disadvantage was associated with a higher mortality rate. We used the Yost Index, a detailed census tract–level measure focused on economic aspects of neighborhood socioeconomic status (SES) [21], to assess disparities with greater precision. Individuals residing in the most disadvantaged group (lowest SES group) experienced higher overall, CVD-specific, and CRC–specific mortality compared with those in the highest SES group. Although disparities in all-cause and cause-specific mortality by SES are well established [36,37], our study is among the first to demonstrate differences in CVD-specific mortality among CRC survivors by neighborhood disadvantage [19]. Leveraging a large, population-based cohort and a multidimensional measure of neighborhood SES, this study captures the spectrum of socioeconomic disadvantage across the U.S. By accounting for competing risks and evaluating multiple mortality outcomes, our findings provide novel insights into the association of neighborhood disadvantage and survivorship outcomes among CRC patients.

### 4.1. Treatment-Related Factors

Chemotherapy for CRC often includes agents such as platinum-based drugs, fluorouracil, and anti-vascular endothelial growth factor (VEGF) inhibitors. While very effective, these drugs are associated with cardiotoxicity [38,39], especially during the early post-treatment periods. Individuals residing in disadvantaged neighborhoods may lack access to specialty care, such as cardio-oncology services, which are often concentrated in academic or high-volume cancer centers located in more affluent areas [40,41]. A prior study linked the SEER data to the American Community Survey and found that poverty, unemployment, and lower income correlated with poor survival outcomes for CRC across different ethnic and racial groups [42]. Conversely, higher levels of education and having commercial insurance were linked to better survival. Lack of specialty care in disadvantaged neighborhoods further contributes to CVD and mortality [40,42]. This limited access may result in delayed identification or inadequate management of treatment-related cardiotoxicities, to preventive services, timely management of treatment-related toxicities, and specialty care [43,44]. Patients from disadvantaged neighborhoods are more likely to receive non-guideline-concordant therapies that elevate CVD risk [45]. Barriers to care, ranging from transportation and financial hardship to fewer local specialists, may further prevent timely treatment or follow-up care [46].

### 4.2. Psychological and Biological Pathways

Our findings suggest that neighborhood disadvantage has an independent impact on CRC survival, which is not fully accounted for by clinical and treatment factors. We observed a clear socioeconomic gradient in the hazard of death of CVD mortality, with individuals in the more disadvantaged neighborhoods associated with a higher risk, even after accounting for competing risks [16].

Chronic exposure to stressors [47,48], such as crime, violence, and limited social cohesion, can activate stress-related biological pathways. These include increased sympathetic system upregulation of proinflammatory signaling via conserved transcriptional gene adversity (CTRA) in leukocytes [49,50,51]. Research in animal and human studies suggests that such changes may promote tumor progression and contribute to excess mortality [52,53]. Integrating social determinants of health into cancer biology research could provide new avenues for intervention, especially when paired with upstream policy efforts to address structural inequities [54].

### 4.3. Physical Environment and Health Behaviors

Neighborhood-level SES also captures features of the built and physical environment that influence health behaviors and exposures. Disadvantaged neighborhoods may lack access to healthy food, safe spaces for physical activity, and adequate healthcare services, factors that extend beyond individual-level characteristics [16]. Such environments contribute to a higher prevalence of obesity, diabetes, tobacco use, unhealthy diet, alcohol consumption, and other shared risk factors for both CRC and CVD [20]. Low CRC screening rates leading to delayed diagnosis are consistently observed in these communities, often resulting in later-stage disease and poorer prognosis [55]. In parallel, persistent inequities in cardiovascular risk factor management and access to timely care for CVD events may contribute to CVD mortality [16]. These findings align with prior research showing that CVD is the leading non-cancer cause of death among CRC survivors [7,20]. Addressing area-level social and structural determinants of health is essential when designing interventions to reduce CVD-specific mortality in this population [56].

### 4.4. Strengths and Limitations

The strengths of this study include the use of a large sample size from a population-based cohort, enabling the examination of differences by neighborhood SES groups across the U.S. population. However, our study has certain limitations. First, while census tract–level SES is a validated proxy, it does not fully capture individual-level socioeconomic variation, which may result in exposure misclassification. Second, neighborhood socioeconomic status (SES) was measured only at diagnosis and may have changed during follow-up. This introduces the possibility of non-differential exposure misclassification, which could bias the results toward the null. Third, the SEER lacks important variables such as insurance status, detailed treatment characteristics, treatment rationale, treatment intensity, prior comorbidities, tobacco use, and alcohol consumption, which may confound the observed associations. Finally, our findings are based on U.S. SEER data and may not be generalizable to populations outside the United States, which have different healthcare systems and demographic characteristics. Despite these limitations, our findings contribute to a better understanding of how neighborhood disadvantage is associated with long-term, cause-specific mortality outcomes in CRC survivors.

## 5. Conclusions

In summary, residence in disadvantaged neighborhoods is independently associated with higher mortality among CRC, with CVD having a significant role in these deaths. Differences in outcomes are influenced not only by individual factors but also by neighborhood-level contextual factors, highlighting the importance of looking beyond the individual level [57]. Future studies that incorporate neighborhood disadvantage into risk stratification could offer a holistic approach essential for advancing precision oncology and guiding effective cancer control interventions. These findings may also inform clinical and public health interventions aimed at reducing cardiovascular risk and improving long-term survival among adults with CRC.

## Figures and Tables

**Table 1:** Characteristics of Colorectal Cancer Patients by Neighborhood Disadvantage (Yost Index Quintiles), SEER 2006–2017 (N + 316,549).

Characteristics n (%)	Group 1 (n = 54,593)	Group 2 (n = 54,995)	Group 3 (n = 58,696)	Group 4 (n = 69,183)	Group 5 (n = 79,082)
Age group, y					
20–49	6572 (12.0)	6438 (11.7)	7095 (12.1)	8415 (12.2)	10,339 (13.1)
50–64	20,264 (37.1)	19,266 (35.0)	20,246 (34.5)	23,664 (34.2)	26,808 (33.9)
65–74	13,937 (25.5)	14,019 (25.5)	14,319 (24.4)	16,568 (23.9)	18,290 (23.1)
≥75	13,820 (25.3)	15,272 (27.8)	17,036 (29.0)	20,536 (29.7)	23,645 (29.9)
Sex					
Female	26,067 (47.7)	26,371 (47.9)	28,130 (47.9)	33,222 (48.0)	37,737 (47.7)
Male	28,526 (52.3)	28,624 (52.1)	30,566 (52.1)	35,961 (52.0)	41,345 (52.3)
Race/ethnicity					
NH-White	26,250 (48.0)	34,846 (63.4)	39,906 (68.0)	49,380 (71.4)	58,999 (74.6)
NH-Black	16,935 (31.0)	8072 (14.7)	5896 (10.0)	5031 (7.3)	3361 (4.2)
Hispanic	8641 (15.8)	8232 (15.0)	7636 (13.0)	6769 (9.8)	5412 (6.8)
NH-Asian/PI	2322 (4.2)	3295 (6.0)	4691 (8.0)	7333 (10.6)	10,383 (13.1)
NH-AI/AN	276 (0.5)	310 (0.6)	271 (0.5)	248 (0.4)	217 (0.3)
Unknown	169 (0.3)	240 (0.4)	296 (0.5)	422 (0.6)	710 (0.9)
Marital status					
Married/Partnered	23,268 (42.6)	27,051 (49.2)	30,759 (52.4)	37,967 (54.9)	47,991 (61.0)
Other	31,325 (57.4)	27,944 (50.8)	27,937 (47.6)	31,216 (45.1)	31,091 (39.0)
Year of diagnosis					
2006–2008	13,074 (23.9)	13,488 (24.5)	14,989 (25.5)	18,357 (26.5)	21,321 (27.0)
2009–2011	13,485 (24.7)	13,865 (25.2)	14,693 (25.0)	17,379 (25.1)	19,743 (24.5)
2012–2014	14,123 (25.9)	13,906 (25.3)	14,555 (24.8)	16,840 (24.3)	18,959 (24.0)
2015–2017	13,911 (25.5)	13,736 (25.0)	14,459 (24.6)	16,607 (24.0)	19,059 (24.1)
Stage					
Localized	21,119 (38.7)	21,669 (39.4)	23,328 (39.7)	28,023 (40.5)	33,817 (42.0)
Regional	20,240 (37.1)	20,611 (37.5)	22,343 (38.1)	26,217 (37.9)	29,788 (37.7)
Distant	13,234 (24.2)	12,715 (23.1)	13,025 (22.2)	14,943 (21.6)	16,107 (20.4)
Tumor location					
Right colon	20,925 (38.3)	21,321 (38.8)	22,923 (39.0)	26,980 (38.9)	30,559 (38.6)
Left colon	15,040 (27.5)	14,813 (26.9)	15,942 (27.2)	18,780 (27.1)	21,226 (26.8)
Rectum	16,747 (30.7)	17,099 (31.1)	18,090 (30.8)	21,521 (31.1)	25,262 (31.9)
Unspecified	1881 (3.4)	1762 (3.2)	1741 (3.0)	1992 (2.9)	2035 (2.6)
Tumor grade					
Well-differentiated	4918 (9.0)	4912 (8.9)	5229 (8.9)	6136 (8.9)	7563 (9.6)
Moderately differentiated	33,051 (60.5)	33,214 (60.4)	35,562 (60.6)	41,902 (60.6)	46,918 (59.3)
Poorly differentiated	6867 (12.6)	7431 (13.5)	8220 (14.0)	10,046 (14.5)	12,093 (15.3)
Undifferentiated or anaplastic	1333 (2.4)	1327 (2.4)	1427 (2.4)	1512 (2.2)	1622 (2.0)
Unknown	8424 (15.4)	8111 (14.7)	8258 (14.1)	9587 (13.9)	10,886 (13.8)
Surgery Receipt					
Yes	44,882 (82.2)	45,973 (83.6)	49,786 (84.8)	59,132 (85.5)	68,166 (86.2)
No	9711 (17.8)	9022 (16.4)	8910 (15.2)	10,051 (14.5)	10,916 (13.8)
Chemotherapy					
Yes	21,428 (39.2)	21,635 (39.3)	23,273 (39.7)	27,510 (39.8)	31,934 (40.4)
No	33,165 (60.8)	33,360 (60.7)	35,423 (60.3)	41,673 (60.2)	47,148 (59.6)
Radiation					
Yes	7927 (14.5)	8085 (14.7)	8597 (14.6)	9884 (14.3)	11,195 (14.2)
No	46,666 (85.5)	46,910 (85.3)	50,099 (85.4)	59,299 (85.7)	67,887 (85.8)
Months of follow-up, Median (IQR)	36 (13–74)	38 (38–79)	41 (16–82)	43 (17–86)	47 (19–90)

Abbreviations: NH—Non-Hispanic; PI—Pacific Islander; AI/AN—American Indian/Alaska Native; IQR—Inter-quartile range. Yost Index Quintiles: Neighborhood socioeconomic status measured using the Yost Index, categorized into quintiles. Group 1 represents the lowest SES, and Group 5 represents the highest SES, with intermediate groups (2–4) representing increasing SES levels.

**Table 2:** All-cause mortality, cause-specific cardiovascular-related mortality, and cancer-specific mortality among US adults 20 years and older, SEER 2006–2017 (N = 316,549).

	Overall Mortality		CVD-Specific Mortality		Cancer-Specific Mortality	
Variable	Hazard Ratio (95% CI)	*p*-Value	Hazard Ratio (95% CI)	*p*-Value	Hazard Ratio (95% CI)	*p*-Value
Yost Index						
Group 5 (highest SES)	Ref		Ref		Ref	
Group 4	1.10 (1.06–1.14)	<0.001	1.07 (0.94–1.22)	<0.001	1.09 (1.06–1.11)	<0.001
Group 3	1.18 (1.14–1.21)	<0.001	1.12 (1.01–1.25)	<0.001	1.15 (1.12–1.19)	<0.001
Group 2	1.23 (1.20–1.26)	<0.001	1.24 (1.14–1.35)	<0.001	1.19 (1.15–1.24)	<0.001
Group 1 (lowest SES)	1.31 (1.28–1.33)	<0.001	1.39 (1.30–1.48)	<0.001	1.24 (1.18–1.30)	<0.001
*p*-trend		<0.001		<0.001		<0.001

Abbreviations: CVD—cardiovascular disease; CRC—colorectal cancer. Models were adjusted for age, sex, race/ethnicity, marital status, year of diagnosis, stage, grade, tumor location, surgery, chemotherapy, and radiotherapy. Yost Index Quintiles: Neighborhood socioeconomic status measured using the Yost Index, categorized into quintiles. Group 1 represents the lowest SES, and Group 5 represents the highest SES, with intermediate groups (2–4) representing increasing SES levels.

**Table 3:** Restricted Mean Survival Time (RMST) by Neighborhood Disadvantage (Yost Index Quintiles).

Yost Index Quintile	RMST (LCL to UCL)	Change in RMST (LCL to UCL)	*p* Value
Group 5 (highest SES)	45.5 (45.3 to 45.6)	Ref	
Group 4	43.7 (43.6 to 43.9)	−1.8 (−1.9 to −1.5)	<0.0001
Group 3	42.8 (42.6 to 43.9)	−2.7 (−2.9 to −2.4)	<0.0001
Group 2	41.7 (41.5 to 41.9)	−3.8 (−4.0 to −3.5)	<0.0001
Group 1 (lowest SES)	40.5 (40.3 to 40.7)	−5.0 (−5.2 to −4.7)	<0.0001

Abbreviations: LCL—Lower Confidence Limit; UCL—Upper Confidence Limit. Yost Index Quintiles: Neighborhood socioeconomic status measured using the Yost Index, categorized into quintiles. Group 1 represents the lowest SES, and Group 5 represents the highest SES, with intermediate groups (2–4) representing increasing SES levels.

## Data Availability

The data analyzed in this study are available from the SEER database, which can be accessed through the NCI (https://seer.cancer.gov/data-software, accessed on 30 September 2024).

## Supplementary Materials

The following supporting information can be downloaded at: https://www.mdpi.com/article/10.3390/cancers17233782/s1, Figure S1: Flow chart of adults 20 years and older with primary colorectal cancer using the Surveillance, Epidemiology, and End Results database (SEER), 2006–2017; Table S1: Joint association between race/ethnicity and neighborhood disadvantage (Yost Index) with mortality; Table S2: Joint association between age and neighborhood disadvantage (Yost Index) with mortality; Table S3: Joint association between gender and neighborhood disadvantage (Yost Index) with mortality.
