# Supplementary material for: Neighborhood Disadvantage and Cardiovascular Mortality Among Colorectal Cancer Survivors
Source: Cancers (Basel). 2025 Nov 26;17(23):3782. doi: 10.3390/cancers17233782 (PMC12691430; doi:10.3390/cancers17233782)
Supplement: Supplementary file 1 [file cancers-17-03782-s001.zip › cancers-3988492-supplementary.pdf]

cancers

MDPI

Supplementary Materials: Neighborhood Disadvantage and Cardiovascular Mortality Among Colorectal Cancer Survivors

Nimish Valvi, Matthew Groenewold, Krista Terracina, Himanshi Verma, Pratibha Shrestha, Kathryn E. Hitchcock, Dejana Braithwaite and Shama D. Karanth

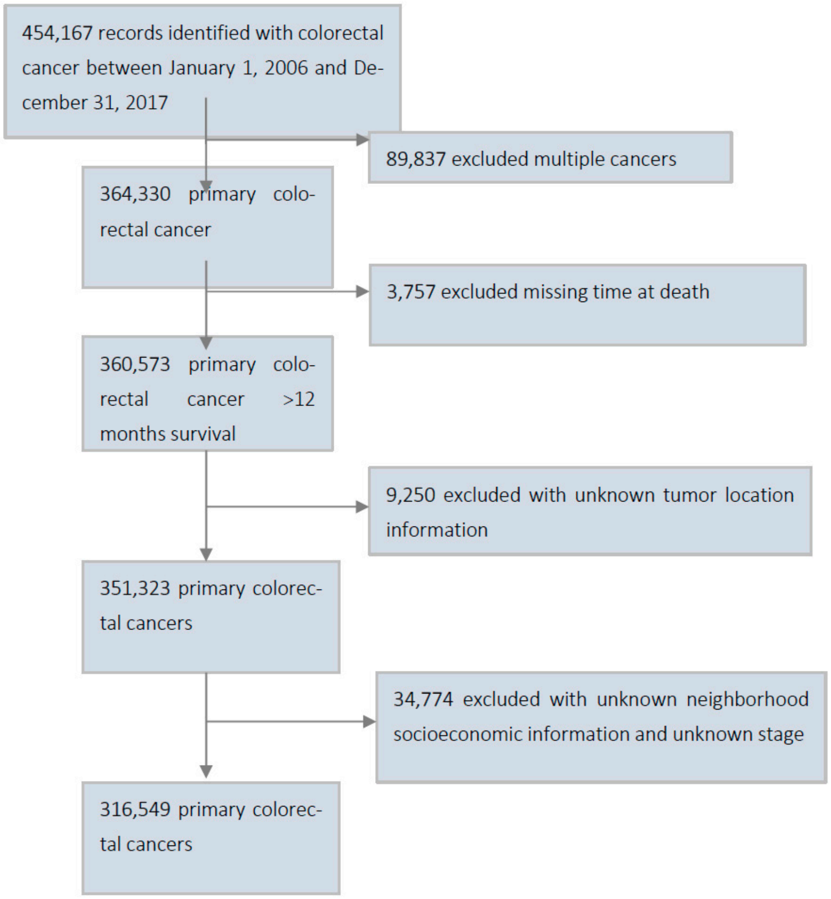

Figure S1. Flow chart of adults 20 years and older with primary colorectal cancer using the Surveillance, Epidemiology, and End Results database (SEER), 2006–2017.

Table S1. Joint association between race/ethnicity and neighborhood disadvantage (Yost Index) with mortality.

SES index (quintiles)	Black vs White	Asian PI vs White	Hispanic vs White
**All cause**			
Group 1 (lowest)	1.00 (0.97–1.03)	0.92 (0.86–0.98)	0.90 (0.86–0.93)
Group 2 (low-middle)	1.00 (0.97–1.04)	1.00 (0.97–1.04)	0.90 (0.87–0.94)
Group 3 (middle)	1.02 (0.98–1.07)	0.94 (0.90–0.99)	0.93 (0.90–0.97)
Group 4 (high-middle)	1.01 (0.97–1.06)	0.92 (0.88–0.96)	0.92 (0.88–0.96)
Group 5 (highest)	1.02 (0.97–1.08)	0.92 (0.89–0.96)	0.97 (0.93–1.02)
	$p < 0.0001$		
**CVD-specific**			
Group 1 (lowest)	0.97 (0.89–1.07)	0.55 (0.43–0.71)	0.72 (0.63–0.83)
Group 2 (low-middle)	0.97 (0.86–1.10)	0.65 (0.52–0.81)	0.65 (0.56–0.76)
Group 3 (middle)	1.03 (0.89–1.19)	0.66 (0.55–0.80)	0.65 (0.56–0.76)

 https://doi.org/10.3390/cancers17233782

Group 4 (high-middle)	0.93 (0.79–1.10)	0.62 (0.53–0.73)	0.77 (0.66–0.89)
Group 5 (highest)	1.07 (0.88–1.30)	0.70 (0.61–0.80)	0.72 (0.60–0.86)
	$p = 0.622$		
**Cancer-specific**			
Group 1 (lowest)	1.04 (1.01–1.08)	1.04 (0.96–1.13)	0.94 (0.90–0.99)
Group 2 (low-middle)	1.04 (0.99–1.09)	0.98 (0.91–1.05)	0.96 (0.91–1.01)
Group 3 (middle)	1.05 (1.00–1.10)	1.01 (0.96–1.08)	0.99 (0.94–1.03)
Group 4 (high-middle)	1.05 (0.99–1.10)	0.96 (0.92–1.01)	0.96 (0.91–1.01)
Group 5 (highest)	1.05 (0.98–1.12)	0.96 (0.92–1.01)	1.03 (0.97–1.09)
	$p = 0.0004$		

Table S2. Joint association between age and neighborhood disadvantage (Yost Index) with mortality.

SES index (quintiles)	50 to 64 vs. 18 to 49 years	65 to 74 vs. 18 to 49 years	75 ≥ vs. 18 to 49 years
**All cause**			
Group 1 (lowest)	1.10 (1.05–1.15)	1.27 (1.21–1.33)	1.49 (1.42–1.56)
Group 2 (low-middle)	1.07 (1.02–1.12)	1.29 (1.23–1.35)	1.54 (1.47–1.62)
Group 3 (middle)	1.07 (1.02–1.12)	1.30 (1.24–1.37)	1.59 (1.51–1.66)
Group 4 (high-middle)	1.06 (1.01–1.11)	1.28 (1.22–1.34)	1.63 (1.56–1.70)
Group 5 (highest)	1.04 (0.99–1.08)	1.31 (1.25–1.37)	1.72 (1.64–1.80)
	$p < 0.0001$		
**CVD-specific**			
Group 1 (lowest)	2.21 (1.69–2.89)	3.87 (2.97–5.06)	5.98 (4.59–7.79)
Group 2 (low-middle)	2.18 (1.60–2.98)	4.20 (3.10–5.71)	7.70 (5.69–10.43)
Group 3 (middle)	2.91 (2.03–4.17)	6.03 (4.24–8.60)	11.40 (8.03–16.17)
Group 4 (high-middle)	2.28 (1.66–3.14)	5.02 (3.68–6.86)	10.49 (7.71–14.27)
Group 5 (highest)	3.18 (2.14–4.73)	7.93 (5.38–11.69)	18.80 (12.81–27.60)
	$p < 0.0001$		
**CRC-specific**			
Group 1 (lowest)	1.01 (0.96–1.06)	1.01 (0.96–1.07)	1.06 (1.01–1.12)
Group 2 (low-middle)	0.99 (0.94–1.05)	1.04 (0.98–1.10)	1.06 (0.99–1.12)
Group 3 (middle)	1.00 (0.95–1.05)	1.03 (0.97–1.09)	1.08 (1.02–1.15)
Group 4 (high-middle)	1.01 (0.96–1.05)	1.02 (0.97–1.08)	1.09 (1.03–1.15)
Group 5 (highest)	0.99 (0.95–1.04)	1.05 (0.99–1.11)	1.11 (1.06–1.17)
	$p < 0.0001$		

Table S3. Joint association between gender and neighborhood disadvantage (Yost Index) with mortality.

SES index (quintiles)	Male vs Female
**All cause**	
Group 1 (lowest)	1.08 (1.06–1.11)
Group 2 (low-middle)	1.08 (1.05–1.11)
Group 3 (middle)	1.09 (1.06–1.12)
Group 4 (high-middle)	1.08 (1.06–1.11)
Group 5 (highest)	1.09 (1.07–1.12)
	$p < 0.0001$
**Cardio specific**	
Group 1 (lowest)	1.41 (1.30–1.53)
Group 2 (low-middle)	1.42 (1.30–1.55)
Group 3 (middle)	1.33 (1.22–1.45)
Group 4 (high-middle)	1.44 (1.33–1.55)
Group 5 (highest)	1.49 (1.38–1.62)
	$p = 0.0246$
**Cancer-specific**	
Group 1 (lowest)	1.03 (0.99–1.06)
Group 2 (low-middle)	1.02 (0.99–1.06)
Group 3 (middle)	1.01 (0.98–1.05)
Group 4 (high-middle)	1.01 (0.98–1.04)
Group 5 (highest)	1.01 (0.98–1.04)
	$p < 0.0001$
